# The Development and Psychometric Characteristics of the “Virtual Identity of Social Media Users” Test

**DOI:** 10.11621/pir.2022.0407

**Published:** 2022-12-30

**Authors:** Dmitry N. Pogorelov, Elena A. Rylskaya

**Affiliations:** a South Ural State University (National Research University), Chelyabinsk, Russia

**Keywords:** Virtual identity, social media, social media users, test, psychometric characteristics, standardization, specification

## Abstract

**Introduction:**

Modern society is characterized by the widespread use of social media, which provides users with communication, leisure, work, and study opportunities. With the growth of such opportunities, more time is being spent online. These circumstances explain why we developed a test entitled the Virtual Identity of Social Media Users (VISCMU).

**Objective:**

To develop and test the psychometric characteristics of the VISMU test.

**Design:**

The research methods included theoretical analysis, modeling, expert assessments, questionnaires, and statistical analysis. The research sample was comprised of 285 users of VKontakte and other social media.

**Results:**

The results of factor analysis proved the acceptability of the three scales identified in the test. Expert assessments showed that the test had sufficient face and content validity. The scales were characterized by optimal indicators of internal consistency, homogeneity, and discriminatory power. The test-retest reliability values demonstrated that the test indicators were stable. Statistically significant differences in the parameter measuring virtual identity in groups with different levels of success in adult life justified a sufficient level of criterion validity. The correlation between the test scales and the components of the factor structure of the modified test “Who am I online?” indicated its compliance with construct validity norms. Positive connections between virtual identity and Internet addiction, smartphone addiction, aggressiveness, hostility, and negative relationships with vitality, indicated sufficient convergent validity. The test has been standardized and specified.

**Conclusion:**

The test was aimed at measuring the extent to which a person’s virtual identity would reveal the specific characteristics of its impact on the individual’s personal development.

## Introduction

Modern life is difficult without the use of the Internet. Since the 1990s, a new virtual socio-cultural space has formed, characterized by wide access to various sources of information. Information openness is becoming one of the defining vectors of social development (Norman, 2017).

Various digital devices mediate mental functions as cultural tools, which leads to the appearance of new activities, the transformation of social interactions, and the formation of new cultural practices. An individual socializing in a digitalized world acquires social experience in online contexts, which leads to people forming a digital persona as part of their personality profile (Soldatova & Rasskazova, 2020).

Currently, social media are one of the most popular online Internet services (Yudina, 2015). These resources are characterized by almost unlimited possibilities: they allow people to communicate and to organize leisure, learning, and work. The massive spread of the Internet in general, and social media in particular, is increasing the time users spend online (Chen, 2013; McNicol & Thorsteinsson, 2017). The current situation, along with the increasing importance of the virtual environment, threatens to reduce the adaptability of individuals to real life and has led to the formation of a special type of ego identity — the virtual identity of social media users (Soldatova & Pogorelov, 2018).

Parents, teachers, and psychologists are alarmed by the increase in the time children and teenagers spend on social media, and the increase in the severity of Internet addictions, especially the need to actively get information from the Internet for educational purposes (Park & Sun, 2017). The global COVID-19 pandemic has also increased the use of the Internet by older generations, which has resulted in the transformation of the subculture of this environment (Chen, Pakpour & Leung, 2020).

Ego identity can be interpreted as the identity and integrity of the personality, the continuity of the “I” in the transformations taking place during personal growth and development (Erickson, 1996). The formation of ego identity is based on generalized childhood identifications, the system of norms and values assimilated by the personality, the attitudes of society, and the expectations and requirements of the peer environment. During the development of consciousness, a person generalizes the experience of past events, which underlies the formation of their system of goals, values, and attitudes. In this context, an increase in the need to reference the virtual space of social media is fraught with the potential for the formation of Internet addiction and decreasing interest in the development of real personal qualities (Moreno, 2019). This subsystem of ego identity—the virtual identity of social media users—dup licates the real identity in virtual space, acting as a modification of the image of the real self. The virtual identity of social media users is compiled from the standardized interface components of the virtual world, aimed at self-presentation on the Internet, and reflects the integrity and identity of the personality in virtual space (Asmolov & Asmolov, 2010).

The analysis of domestic and foreign studies on the problem of virtual identity served as the basis for our development of a conceptual model of the virtual identity of social network users. The virtual identity structure has three components.

The first of them, “cyberaddiction,” reflects the individual’s level of reference to social networks, as well as the emergence of addictive tendencies with excessive use of social networks (Kochetkov, 2020; Kuss, 2021; Rajesh & Rangaiah, 2020). The main prerequisite for the development of cyberaddiction is the incomplete resolution of age-related crises, which manifests itself in the development of an identity crisis. The Internet environment is attractive for resolving this crisis since it allows the user to construct a desired reality in it. Unresolved problems of aging, which serve as criteria-based grounds for the formation of an alternative to a person’s real identity, indicate a lack of human vitality and actualize various kinds of dependencies, including, probably, cyberaddiction. Virtual “friends,” “likes,” and “posts” are becoming more valuable than communication in a real environment, which reflects the high reference of social networks. Moreover, cyberaddiction is characterized by an increase in tendencies for aimless activity on the Web.

The second component, “acceptance of subculture,” characterizes the degree of approval by the individual of the specific norms adopted in virtual communities (Kurbatov, Volkov, & Vodenko, 2019; Senchenko, 2016). The virtual personality creates a new virtual culture, which becomes part of the culture as a whole. New forms of mass collective behavior of users on social networks act as a kind of electronic frontier on which users can oppose traditional reality that does not suit them for reasons of justice, morality, order, and values. The most striking manifestation of the subculture of social network users is the potential for user anonymity , which does not require providing true personal data on social networks, and thus reduces moral barriers in communication, and permits manifestations of aggressiveness, including cyberbullying. It is also impossible not to note the ratio of obscene vocabulary, images of a “beautiful life,” and tolerance for punctuation and grammatical errors.

The third component, “virtual image,” reflects a complex representation of the individual’s physical and psychological properties as projected in virtual space, as well as the possibilities and advantages of communication in social networks (Luchinkina, 2016; Zekeryaev, 2019). This image has no real content, but consists solely of signs-symbols, including the physical and psychological properties of the individual, and actions unfolding within the framework of Internet communication. Since the user is not physically present in the virtual space of social networks, his actions can be implemented exclusively through virtual communication.

The foregoing determines the importance of adequately measuring the virtual identity of social media users. However, since this phenomenon is new and insufficiently studied, there are no special psycho-diagnostic tools for studying aspects of virtual identity. Such a tool could aid in preventing excessive Internet use and solving the problem of the negative impact of Internet content on personal development and interpersonal interaction of people of all ages. The purpose of our study was to develop and test the psychometric characteristics of the Virtual Identity of Social Media Users (VISMU) test by:

1. Conceptually justifying and developing the structure of the VISMU test to measure the virtual identity of social media users.
2. Checking the main psychometric characteristics of the test: face and content validity, reliability, homogeneity, discriminatory power, and criterion and construct validity.
3. Standardizing the VISMU test.
4. Giving a descriptive specification of the test.

## Method

Our research was based on the methods of theoretical analysis, synthesis and generalization, induction and deduction, modeling, expert evaluation, and questionnaires. Descriptive statistics, factor analysis (principal component analysis (PCA) followed by Varimax rotation), Kendall’s W, Cronbach’s α, Spearman-Brown’s *rt*, Ferguson’s σ, Pearson’s *r*, Mann–Whitney’s U, Kolmogorov-Smirnov’s *d*, and Student’s *t* criteria were used for mathematical processing of the data. The results were processed using the IBM SPSS Statistics v. 26.0 and MS Excel statistical analysis suites.

### Participants

The research sample was comprised of 285 users of VKontakte and other social media, age 18 to 72 (X = 37.49, SD = 13.61). The sample involved 197 women (69.1%) and 88 men (30.9%), including university students, and representatives of the blue and white collar professions. The procedures of factor analysis and verification of the psychometric characteristics of the test were carried out on this sample.

To confirm the criterion validity, a content analysis of the pages of users of an additional sample (N = 30) on VKontakte and other social media was carried out. This type of validity is based on comparing test indicators with data obtained on the basis of objective (external) criteria. Such criteria can be success in adult life, such as personal and professional self-determination, creating a family, and performing pro-social activities (Ananiev, 2001). The first group (N = 15) included subjects who currently had no families, had no job, showed signs of alcoholism, or use obscene language. The second group (N = 15) included subjects who created families, had a profession, and showed no signs of obscene language, deviant, or aggressive behavior.

The test was standardized using an additional sample of 495 people, including 231 men (46.7%) and 264 women (53.3%), age 18 to 57 (X = 34, 96, SD = 10.35). The sample included students in secondary and higher educational institutions, teaching staff (educators and heads of preschool educational institutions, teachers and headmasters, and lecturers at secondary and higher educational institutions), representatives of business (individual entrepreneurs), service professionals (cleaners, storekeepers, drivers), and the unemployed. In this regard, we can consider this sample to be representative.

The data was collected and processed between 2017 and 2021.

### Procedure

During the first stage, using theoretical analysis, we developed a conceptual model of the structure of the virtual identity of social media users, which included three components: Cyberaddiction (CA), Acceptance of Subculture (AS), and Virtual Image (VI) (Pogorelov & Rylskaya, 2021). In the second stage, we selected methods to diagnose each of the identified structural components, formed a data base for the research, and developed test statements (initially 103 items). The third stage included factor analysis, for which we selected the questions with the maximum factor load. We then verified the face and content validity of the VISMU test. As a result, we formed a version of the VISMU methodology which includes 43 items and the three scales: CA, AS, and VI. Further, we determined the psychometric characteristics of the VISMU test (internal consistency, test-retest reliability, criterion and construct validity), and standardization, taking into account that women and men do not differ in the structural components of their virtual identity; however, we found and took age differences into account.

## Results

### A conceptual model of the virtual identity of social media users

The conceptual foundations for the development of the VISMU test are the understanding of virtual identity as an integral phenomenon, which is a subsystem of ego identity coexisting with the structure of real identity, consisting of the textual, visual, and audio characteristics of a virtual image, and reflecting the physical and psychological properties and communication features which determine the integrity and identity of the personality within the subculture of social media users (Rylskaya & Pogorelov, 2021). Virtual identity is understood from the standpoint of the relationship between the concepts of ego identity and vitality at two points of contact: normative crises and life tasks (Rylskaya, 2005; Soldatova, 2005). To prepare the test, we used the technology developed by Baturin, which has been actively used by representatives of the Chelyabinsk School of Psychology (Baturin & Melnikova, 2009).

We described the conceptual model of the virtual identity of social media users in our previous publications (Pogorelov, 2020; Rylskaya & Pogorelov, 2021). In characterizing virtual identity, we distinguished the three components.

1. Cyberaddiction (CA) is considered an obsessive desire to use the Internet or to spend much time on the Internet (Kochetkov, 2020; Kuss, 2021; Rajesh & Rangaiah, 2020).
2. Acceptance of Subculture (SA) is the degree of a person’s approval of the special norms, rules, and values characteristic of the virtual space of social media (Kurbatov, Volkov & Vodenko, 2019; Senchenko, 2016).
3. Virtual Image (VI) ensures the integrity and identity of the personality in the social media space and reflects the desired image of its creator (Luchinkina, 2016; Zekeryaev, 2019).

According to this conceptual model of the virtual identity of social media users, we created a databank for the development of test statements. The bases for the test design were the scales of standardized psycho-diagnostic techniques (Lee and Quigley’s scale for measuring self-presentation tactics; Thomas’ questionnaire “Style of behavior in conflict;” Sukhikh and Korytchenkova’s test of the moral and ethical characteristics of a person and the level of their psychoethical development; Mikhelson’s communication skills test; Morosanova’s questionnaire “Behavioral self-regulation style;” and the method for diagnosing addictive identity developed by Dmitrieva, Perevozkina, Perevozkina, and Samoilik). When formulating the test statements, we both used separate items to diagnose the components of the structure of virtual identity, and developed new items; this was to ensure that the phenomenon of the virtual identity of social media users was taken into account as precisely as possible. As a result, we compiled 103 items for the original version of the VISMU test.

### Factor analysis of the test items

At the next stage, we carried out a factor analysis to select the items with the maximum factor load. An analysis of the eigenvalues for the items allowed us to identify three factors. After the rectangular Varimax rotation, we selected the items with the maximum factor loads. At this stage, we analyzed all the test items and excluded those with unsatisfactory psychometric characteristics. The selected factors accounted for 75.6% of the variance. The items obtained as a result of factorization formed a version of the VISMU test, which measured the three scales: CA, AS, and VI.

CA was formed by statements about the highly subjective significance of social media, which is often even more significant for users than the real world. This factor served as a content-related confirmation of the conceptual model.

AS included variables demonstrating the level of changes in the user’s behavior or opinion under the influence of the norms accepted in virtual communities. The set of items (variables) of this factor corresponded to the hypothetical scale.

VI described attitudes towards social media presentation and included characteristics linked with the ability to more flatteringly present one’s physical characteristics and personality traits, as well as the benefits and opportunities of online communication.

Further, we identified the test items with the maximum factor load (no less than 0.4). The items obtained during the factor analysis formed the basis of the original version of the VISMU test; during the repeated factorization, we obtained a reproducible structure.

Then, this test version was presented to a sample of 285 people simultaneously with the original version. The results were above the critical values (*Table 1*).

**Table 1:** Correlation coefficients between the scales of the VISMU test versions

The scale of the VISMU test	Correlation coefficients r
CA	0.721**
AS	0.837**
VI	0.734**

** — the correlation is significant at the level of 0.01

We found that all the selected items made the largest contributions to the corresponding factors and the smallest contributions to the other factors (*Table 2*). In this regard, we justified the optimality of the selected 3-factor model.

**Table 2:** Factorization of the VISMU test items

Statements of the VISMU test	Factors/Loads		
	1	2	3
I often photoshop my pictures before posting them on the Internet;	0.045	–0.220	**0.887**
I prefer being online to being intimate with a partner;	**0.844**	–0.248	–0.095
I think insults and foul language on the Internet are a matter of course;	0.038	**0.663**	–0.163
I enjoy watching pictures or videos on the Internet rather than reading a text;	**0.793**	–0.071	0.019
I believe you should not post photos on social media showing flaws in appearance;	–0.122	–0.204	**0.926**
The Internet allows me to express myself;	0.156	**0.499**	–0.221
I communicate on the Internet with people from other cities or countries;	0.074	**0.920**	–0.185
Sometimes I use social media without intending to communicate with anyone;	**0.861**	0.089	0.023
I feel guilty when I realize that my image in the real world is different from my social media image;	0.163	–0.435	**0.795**
I exaggerate the significance of my achievements on the Internet;	–0.017	–0.333	**0.867**
I fail to reduce my time online;	**0.884**	0.051	0.245
I like that I can fully express myself on the Internet;	0.186	**0.867**	–0.139
Sometimes I give incorrect information about my identity on the Internet;	0.176	**0.884**	–0.029
I post pictures of important events in my life on social media;	**0.806**	0.460	0.039
I do not always indicate my true identity, such as name, picture, location, when registering on social media;	0.299	**0.527**	–0.214
I believe social media posts allow me to convey my thoughts to many people;	**0.926**	0.092	–0.043
I am a member of Internet groups dedicated to an ideal appearance;	0.049	0.032	**0.919**
I often visit profiles of strangers on social media;	0.150	**0.503**	–0.177
I believe it is not necessary to follow the rules of the Russian language on the Internet;	0.024	**0.538**	–0.150
Unfortunately, the merits of a person in the real world often remain unrecognized, no matter how hard they try;	**0.815**	–0.074	0.138
I make the impression of a successful and attractive person on social media;	–0.042	–0.032	**0.940**
Playing online or visiting social media helps me to change my mood;	**0.841**	0.043	0.093
I feel empty, depressed, and irritated when I’m not connected to the Internet;	**0.860**	0.272	0.202
It is much more convenient to make purchases on the Internet;	0.167	**0.883**	0.017
I believe my image should be perfect on the Internet;	0.147	–0.185	**0.901**
The first thing I do when I wake up is check my email and open social media pages;	**0.888**	0.257	0.082
I believe it is quite conceivable to take on a different persona on the Internet;	–0.006	**0.929**	–0.032
The Internet allows me to get rid of boredom;	**0.782**	0.076	0.006
Internet users think I am more professional than I really am;	0.130	–0.044	**0.939**
I believe I look younger and more attractive in pictures and videos on the Internet;	0.061	–0.029	**0.891**
I have entered into conflicts on social media without identifying my real identity;	0.173	**0.874**	–0.038
I have alternative pages on social media where I take on a different persona;	0.378	**0.829**	–0.227
I think I look more impressive in pictures than in reality;	0.252	0.132	**0.892**
If I have a bad opinion of a person and do not like their behavior on the Internet, I hardly try to hide it from them;	0.051	**0.876**	–0.257
I notice that the time I spend on the Internet is increasing;	**0.823**	0.144	0.020
Internet users think I am smarter than I really am;	0.304	–0.064	**0.866**
I do not go to the library, as it is easier for me to find any information on the Internet;	0.236	**0.817**	–0.181
I often scroll through the news feed on the social media aimlessly;	**0.898**	0.229	0.091
Sometimes I feel an overwhelming urge to refresh a page on social media;	**0.867**	0.308	0.087
It happens that I insist on my own way when discussing on the Internet, even when I am not sure that I am right;	0.064	**0.950**	–0.076
My mood improves when I use the Internet;	**0.814**	0.193	0.095
I am sure to highlight my achievements and success on social media;	**0.730**	0.172	0.145
I aimlessly browse other people’s pages on social media.	**0.790**	0.154	–0.030

Factoring method: principal component analysis. Rotation method: Varimax with Kaiser normalization. Rotation converged in 9 iterations.

The factor loading values higher than |0.4| are highlighted in bold and indicated as the significant loadings for the corresponding factor.

### Face and content validity

At the next stage, we determined the face validity of the test to assess the degree of understanding of the test content by people who did not know about the social networks where our study was performed. They took part in the assessment of the face validity evaluating the items according to the criteria of “specificity,, “literacy,” and “understandability.” To measure the concordance of expert opinions, we calculated Kendall’s concordance coefficients was presented in *Table 3* for the three scales. As a result of analyzing the expert opinions, we reformulated six test items marked as insufficiently specific or understandable.

Further, we determined the content validity to highlight the representativeness of the content of the test items. To do this, the VISMU test was evaluated by experts to determine its compliance with the subject of our diagnostics — the structural components of the virtual identity of social media users. Three experts participated in the evaluation: one PhD in psychology, one PhD in pedagogy, and a practicing psychologist. The experts were experienced in theoretical and empirical research and practical work on virtualization and the impact of the Internet on personal development. Based on the expert assessments, we adjusted five items in the test which were not formulated specifically enough and were not sufficiently substantial.

**Table 3:** Concordance of the expert opinions on the scales of the VISMU test

The scale of the VISMU test	Kendall’s concordance coefficients W	
	Non-professional experts	Professional experts
CA	0.59**	0.73**
AS	0.71**	0.73**
VI	0.81**	0.82**
Integrated index	0.68**	0.75**

** — the coefficient of concordance is significant at the level of 0.01 (value χ^2^)

Thus, we selected and adjusted 43 items of the VISMU test, or 42% of the initial 103 items.

At subsequent stages, we determined the reliability, homogeneity, discriminatory power, and criterion and construct validity, and performed standardization and specification.

To determine the main psychometric characteristics of the test, the respondents were tested using the final version of the VISMU test, the modified Kuhn-McPartland test, Young’s “Internet addiction” test (adapted by Loskutova), a short version of Sheinov’s questionnaire “Smartphone Addiction Scale,” Bass and Darka’s questionnaire for studying the level of aggressiveness (adapted by Khvanov, Zaitsev, and Kuznetsova), and Rylskaya’s “Human vitality” test. To determine criterion validity, we carried out a content analysis of the pages of users from an additional sample (N = 30).

### Reliability, homogeneity, and discriminatory power

To determine the test’s reliability as an indicator of its stability concerning measurement errors, we calculated Cronbach’s α for its final version. To identify the test’s internal homogeneity, we split the scales into two parts. Then, we calculated the Spearman-Brown coefficient. We determined the discriminatory power as the ability of the test to differentiate the subjects from the minimum to the maximum result by calculating Ferguson’s σ. The results showed satisfactory indicators of the internal consistency, homogeneity, and discriminatory power of the VISMU test (*Table 4*).

**Table 4:** Indicators of reliability, homogeneity, and discrimination power of the VISMU test

The scale of the VISMU test	Cronbach’s reliability coefficient σ	Spearman-Brown ho-mogeneity coefficient rt	Ferguson’s discrimination coefficient σ
CA	0.976	0.854	0.938
AS	0.967	0.835	0.946
VI	0.979	0.939	0.943

Further, we determined the coefficients of the correlation between the test scales, presented in *Table 5*. We found that the structural components of virtual identity were moderately correlated with each other. The results allowed us to assume that the nature of virtual identity is integral, given a certain autonomy and qualitative uniqueness of its individual components.

**Table 5:** Correlation of the scales of the VISMU test

The scale of the VISMU test	CA	AS	VI	Integrated index
CA	–	0.302**	0.169**	0.826**
AS	0.302**	–	0.274**	0.505**
VI	0.169**	0.274**	–	0.528**
Integrated index	0.826**	0.505**	0.528**	–

** — the correlation is significant at the level of 0.01

### Test-retest reliability

We calculated the test-retest reliability to assess the stability of the VISMU test indicators during repeated measurements. To this end, we twice tested the participants of advanced training courses of the State Budgetary Institution of Additional Professional Education Chelyabinsk Institute of Professional Development and Retraining of Educators (N = 92) using the VISMU test. The interval between the tests was three weeks. The correlation indicators between the results of the first and second tests demonstrated high test-retest reliability, which indicated the stability of the VISMU test as to time and, consequently, the stability of the virtual identity of social media users over time (*Table 6*).

**Table 6:** Indicators of the test-retest reliability of the VISMU test

The scale of the VISMU test	Correlation coefficient r
CA	0.945**
AS	0.963**
VI	0.956**

** — the correlation is significant at the level of 0.01.

### Criterion validity

This type of validity is based on comparing test indicators with data obtained on the basis of objective (external) criteria using the extreme group approach. Based on the theoretical analysis, such criteria can be the success in adult life — namely, personal and professional self-determination, creating a family, and performing pro-social activities (Ananiev, 2001). These tasks can be carried out when performing such social roles as citizen, family member, or professional (Rylskaya, 2013). For the formation and harmonious development of a mature personality in the real world, it is necessary to successfully solve the main life tasks of this period in life. Successfully solved age-related tasks are correlated with weakly expressed virtual identity. Low success rates in solving the main life tasks of the period of maturity (lack of a family, a permanent place of work, a tendency to addictive behavior) are associated with dissatisfaction with life in the real world. These characteristics lead to a pronounced virtual identity.

As a result, two samples were formed based on the content analysis of the pages of the social media users, which took note of their personal semantic units (the content of the profile, topics of posts, content of visual and textual information) and universal semantic units (having a family, having a job, the presence of obscene language, demonstrating deviant and aggressive behavior). The first group (N = 5) included subjects who currently had no families or job, showed signs of alcoholism, or used obscene language. The second group (N = 15) included subjects who created families, had a profession, and showed no signs of obscene language, deviant, or aggressive behavior. As a result of applying the Mann-Whitney U criterion, we obtained statistically significant differences between the two groups of respondents in terms of the integral indicator of virtual identity (U = 33, p < 0.01), as well as in all scales, in particular, CA (U = 34 , p < 0.01), AS (U = 39, p < 0.01), and VI (U = 36, p < 0.01).

Accordingly, those users who were assigned to the group with high rates of success in adult life were found to have the lowest rates of virtual identity in general as well as on individual scales, which significantly differed from the group with low rates of success in adult life. Users who were assigned to the group with high rates of success in adult life were less prone to Internet addiction, more critical of the specific norms of the subculture of social network users, and less prone to idealization of their virtual image.

*The Contextual (concept) validity* of the test was based on a factor analysis of the results of the modified Kuhn-McPartland’s test, with the subsequent calculation of correlations with the scales of the VISMU test. This method highlights the substantive features of the identity revealed through the use of the respondents’ answers to the question “Who am I?” through self-descriptions of the personality. Accordingly, the modification of the “Who am I online?” method assumes the respondents’ answer to the corresponding question regarding the virtual environment. The primary processing of the Kuhn-McPartland’s test included the conversion of qualitative data represented by identification characteristics into quantitative indicators by the coding data. The factor analysis of the results of the PCA (principal component analysis) followed by Varimax rotation allowed us to identify the factor structure correlated with the previously identified structure of the virtual identity of social media users.

The first factor included self-descriptions related to the specific features of their social media activities (“user”), which were often aimless (“gamer,” “music addict”) and reflected the high importance of social media for them (“writer,” “reader”). This factor also included self-descriptions of emotional states arising from these activities (“cheerful,” “joyful,” “inspired,” “delighted”). Notably, positive emotions arising from social media activities can reinforce the tendencies toward cyberaddiction described within the factor structure of the virtual identity of social media users. This factor is called Virtual Activity and is correlated with the CA factor.

The second factor involved self-descriptions reflecting the norms accepted in the virtual space of social media: an aggressive style of behavior (“harsh,” “evil”), a tendency to conceal information about oneself and hide behind the associated anonymity (“deceptive,” “opposite”), and the desire to attract attention and openness in communication (“demonstrative,” “critic”). This factor was called “Norms of behavior in social media” and correlates with the AS factor.

The next factor described the specific features of the user’s self-presentation on social media. This factor included a self-description of the virtual image (“user picture,” “depicted”) at the level of physical (“fashionable,” “beautiful”) and psychological properties (“sociable,” “active,” “open-minded”). This factor included idealized self-descriptions (“ideal,” “bright”). The factor included self-descriptions demonstrating the particular features of the sphere of communication and interaction with people (“sociable,” “friend,” “open-minded”), as well as the ratio of the real and virtual image (“real,” “as in life,” “true,” “different”). This factor was called “Virtual self-presentation” and correlated with the VI component.

Next, we calculated the correlations between the scales of the VISMU test and the components of the factor structure of the modified Kuhn-McPartland test (*Table 7*). The correlation coefficients were found to be satisfactory, which indicated the optimal level of construct validity and simultaneously emphasized the uniqueness of the VISMU test.

**Table 7:** Correlations between the scales of the VISMU test and the components of the factor structure of the modified Kuhn-McPartland test

The scale of the VISMU test	Component of the factor structure of the modified Kuhn-McPartland test	Correlation coefficient r
CA	Virtual activity	0.524**
AS	Norms of behavior in social media	0.457**
VI	Virtual self-presentation	0.431**

** — the correlation is significant at the level of 0.01.

For *convergent validity*, the virtual identity of social media users diagnosed by the questionnaire should have positive correlations with Internet addiction, smartphone addiction (Luppicini, Alotaibi, 2021; Sheinov, 2020), and aggressiveness and hostility (Terizi, Chatzakou, Pitoura, Tsaparas, & Kourtellis, 2021), and negatively correlate with vitality (Rylskaya, 2016). To establish convergent validity, we used several psychodiagnostic techniques: to determine Internet addiction—Young’s test “Internet addiction” (adapted by Loskutova); to identify smartphone addiction—a short version of Sheinov’s questionnaire “Smartphone Addiction Scale”; to diagnose the level of aggressiveness and hostility—Bass and Darka’s questionnaire for studying the level of aggressiveness (adapted by Khvanov, Zaitsev, and Kuznetsova); and to identify the level of vitality—Rylskaya’s test “Human vitality.” The results of the correlation analysis are presented in *Table 8*.

**Table 8:** Correlations between the virtual identity of social media users and its components and conceptually related mental properties

The scale of the VISMU test	Internet addiction	Smartphone addiction	Aggressiveness	Hostility	Vitality
CA	0.703**	0.601**	0.375**	0.332**	-0.574**
AS	0.312**	0.404**	0.511**	0.401**	-0.291*
VI	0.366**	0.303**	0.115*	0.141*	-0.52**
Integrated index	0.759**	0.71**	0.52**	0.465**	-0.685**

* — the correlation is significant at the level of 0.05; ** — the correlation is significant at the level of 0.01

The results demonstrated the presence of significant positive correlations between virtual identity and its components with Internet addiction, smartphone addiction, aggressiveness, and hostility, as well as significant negative correlations of virtual identity and its components with vitality, which indicates that the test has sufficient convergent validity.

*The test was standardized* using a sample of 495 people, including 231 men (46.7%) and 264 women (53.3%) age 18 to 57 (X = 34, 96, SD = 10.35). The sample included students from secondary and higher educational institutions, teaching staff (educators and heads of preschool educational institutions, teachers and headmasters, lecturers of secondary and higher educational institutions), representatives of business (individual entrepreneurs), service professions (cleaners, storekeepers, drivers), and the unemployed. In this regard, we can consider this sample to be representative.

The check of the distribution type of the test scores (Kolmogorov-Smirnov’s d test) according to the final VISMU test version showed that the empirical distributions for the indicator had a normal form (p>0.05). The results obtained using a Student’s t test allowed us to assert that women and men do not differ in their degree of expressing their virtual identity. Therefore, to standardize the results and build norms, we did not take gender into account. However, we found age differences for some components of virtual identity, namely, according to the CA and VI scales.

At ages under 35 years, the indicators of cyberaddiction (t = 2.534; p = 0.05) and virtual image (t = 2.642; p = 0.05) were significantly higher. Age differences in the degree of expressing virtual identity were apparently linked with the fact that these resources are more actively used by young people, for whom social media is more referential. We divided the sample into two subgroups: younger and older than 35, because, according to Strauss and Howe’s generational theory (Howe & Strauss, 1991), people under 35 (representatives of generations Y and Z) are most susceptible to the influence of digitalization, since their socialization from an early age was connected with the spread of the Internet.

**Table 9:** Mean values and standard deviations for the virtual identity of social media users and each of its components

M±σ	Integrated index	CA	AS	VI
Users aged from 18 to 35	143.92±26.62	57.83±9.25	48.18±8.91	37.9±7.15
Users aged from 36 to 57	133.73±24.64	53.74±8.6	47.25±8.62	32.74±6.37

During the standardization procedure, we calculated mean values and standard deviations for the virtual identity of social media users and each of their components (*Table 9*). They were converted to a standard 10-point sten scale (*Table 10*). The number of points scored according to the integrated index from 1 to 3 corresponded to a low level of virtual identity, from 4 to 7 to a medium level, and from 8 to 10 to a high level. The levels of manifestation for the components of virtual identity were determined similarly.

**Table 10:** Recalculation of raw scores of the VISMU test into stens

Stens	1	2	3	4	5	6	7	8	9	10
Levels	Low level			Medium level				High level		
Users from age 18 to 35	CA									
	≤39	40–	45–	50–	54–	59–	63–	68–	73–	≥77
		44	49	53	58	62	67	72	76	
	AS									
	≤30	31–	36–	40–	45–	49–	54–	58–	63–	≥67
		35	39	44	48	53	57	62	66	
	VI									
	≤24	25–	28–	32–	35–	39–	42–	46–	50–	≥53
		27	31	34	38	41	45	49	52	
	Integrated index									
	≤91	92–	105–	118–	132–	145–	158–	171–	185–	≥198
		104	117	131	144	157	170	184	197	
Users from age 36 to 57	CA									
	≤37	38–	42–	46–	50–	55–	59–	63–	68–	≥72
		41	45	49	54	58	62	67	71	
	AS									
	≤30	31–	35–	40–	44–	48–	53–	57–	61–	≥65
		34	39	43	47	52	56	60	64	
	VI									
	≤20	21–	24–	27–	31–	34–	37–	40–	43–	≥46
		23	26	30	33	36	39	42	45	
	Integrated index									
	≤84	85–	98–	110–	122–	135–	147–	159–	172–	≥184
		97	109	121	134	146	158	171	183	

### Processing of the results

To calculate points for each of the scales and the integrated index, scores from 1 to 5 were assigned to the answers to the test items according to the Likert scale (from 1 = strongly disagree to 5 = strongly agree). The indicators were summed for each scale and the integrated index of virtual identity. The Appendix contains the instructions, stimulus material, the answer sheet, and the key for processing the results.

## Discussion

The design of the VISMU test assumed the construction of a conceptual model of the structure of virtual identity which included three components: Cyberaddiction (CA), Acceptance of Subculture (AS), and Virtual Image (VI). Based on the empirical data and using factor analysis, we showed that distinguishing the corresponding scales in the VISMU test is justified.

When checking face and content validity, based on the results of the expert assessment (non-professional and professional experts), we adjusted some items, which allowed us to increase the degree of their content-richness and specificity.

We determined that the scales of the VISMU test were characterized by sufficient indicators of internal consistency (α = 0.967–0.979), homogeneity (rt = 0.835–0.939), and discriminatory power (σ = 0.938–0.946). The test-retest reliability check showed satisfactory results (r = 0.945–0.963).

When determining criterion validity, a comparison of the extreme groups according to the criterion of success in adult life showed statistically significant differences in the parameter of the extent of virtual identity (U = 33, p < 0.01).

When justifying construct validity, we faced difficulties connected with the insufficient development of the concept of the “virtual identity of social media users” in the literature, on the one hand, and in choosing diagnostic tools to measure the indicators with which the components of virtual identity could be correlated, on the other. When analyzing the diagnostic tools, it seemed to be most appropriate to use the modified Kuhn-McPartland test, as it allowed us to diagnose the features of virtual identity based on personality self-description. The structure revealed on the basis of the factor analysis was correlated with the factor structure of the VISMU test. These correlations between the scales of the developed test and the components of the factor structure of the modified Kuhn-McPartland test were satisfactory (r = 0.431–0.524), which indicates the construct validity of the VISMU test.

When checking convergent validity, we relied on the conceptual model of virtual identity, according to which there are positive connections between virtual identity and its components with Internet addiction (r = 0.312–0.759), smartphone addiction (r = 0.303–0.71), aggressiveness (r = 0.115–0.52) and hostility (r = 0.141–0.465), and negative connections with vitality (r = –0.291– –0.685). The correlations indicated the convergent validity of the test.

The standardization of the VISMU test involved the calculation of the mean values and standard deviations for virtual identity and each of its components. The data were converted into a standard 10-point sten scale. When analyzing the data, we found no significant differences for gender; however, there were differences for age. At ages under 35 years, the components CA and VI were significantly higher, which was taken into account to standardize the VISMU test.

When developing the test specification, we described the interpretation of the test results in general, as well as for the scales separately using three indicators: high, medium, and low.

The VISMU test enriches the range of diagnostic tools that can be used in theoretical and applied research on personal development in the digital world. The VISMU test can be used in practical psychology when organizing preventive work on the problems of Internet addiction, and reducing the negative impact of virtual content on personal development in representatives of all age groups.

At the same time, we note that in our study, cyberaddiction acted as one of the components of virtual identity. This leads to the fact that the severity of virtual identity acquires a negative meaning, is associated with cyber-aggression, and the low ability for self-control and low vitality. Meanwhile, studies on the social identity of the individual (in its organizational, ethnic, civil, and universal aspects) indicate the inconsistency of this phenomenon, and its multidirectional influence on the individual and social interaction. In this regard, it seems appropriate to consider not only the severity, or strength of identification, but also its valency, that is, an individual’s assessment of his belonging to a social category.

We believe that in future studies, clarifications are needed which take into account the motives for users to access social networks, as well as the nature of personal requests, personal expectations, and subjective preferences in network interaction. We also suggest that the analysis of the identified phenomenon will be more meaningful if, with the help of cluster analysis, various types of virtual identity are distinguished, differing in the degree of expression of its three indicators.

In the future, we plan to continue work on checking the test validity (construct, competitive, and environmental), and study the social desirability of the respondents’ answers. Another promising area of study is the specifics of the virtual identity of users of different social media, including those which are currently gaining popularity (for example, Tik-Tok). We also plan to study the connections between virtual identity and the personal characteristics of social media users, and regional and national characteristics.

Turning to the limitations, further plans, and prospects of the study, we note the importance of expanding the standardization sample of the test to clarify the levels of virtual identity severity. We also note the need for a more even distribution of respondents according to the qualitative characteristics inherent in the general population.

## Conclusion

We developed the VISMU test and analyzed its psychometric characteristics. The use of the test revealed the extent of the structural and content components of the virtual identity of social media users. Distinguishing the indicators of virtual identity during diagnostic procedures will reveal the presence of virtual identity in the personality structure, determine its components, and reveal their influence on personal development.

High scores on the VISMU test were connected with the significance of the virtual space for the user, spending time on social media aimlessly, a high degree of acceptance of the norms of social media users’ subculture, and the creation of an idealized virtual image. Users with high levels of virtual identity were Internet-addicted, prone to smartphone addiction, aggressive, and less active. Their expressed virtual identity performed a compensatory function for activity in real life.

The theoretical basis of the test was a conceptual model of the virtual identity of social media users based on an analysis of domestic and foreign sources on this problem. We proved that it is optimal to identify the CA, AS, and VI scales in the VISMU test. These scales were characterized by satisfactory indicators of face and content validity, internal consistency, homogeneity, and discriminatory power, as well as test-retest reliability, and meet the norms of criterion and construct validity. The VISMU test is a standardized psycho-diagnostic tool. Within the development of the test specification, we described a detailed interpretation of the results.

## Limitations

We note the importance of using more precise constructs in assessing the criterion validity of the VISMU test. The sample of the test standardization could also be extended to specify the manifestation levels of the indicators of the social media users’ virtual identity. The sample of the test standardization could also be extended to specify the levels of manifestation of the integral indicator of virtual identity and its three components A more detailed interpretation of the scales of the VISMU test is also needed. These issues will be studied in our future research.

## Appendix

### Тест «Виртуальная идентичность пользователей социальных сетей («ВИПСС»)»

*ФИО_________________________________________ Возраст _________________________*

*Дата________________________________________ Пол _______________________________*

*Адрес электронной почты _____________________________________________________________*

*Инструкция*: Перед Вами утверждения, касающиеся тех или иных аспектов поведения, общения, а также Вашего образа в социальных сетях. Опираясь на шкалу ответов от 1 до 5, выразите степень Вашего согласия с каждым из утверждений, приведенных ниже. Степень Вашего согласия соответствует следующим показателям:

1 — совершенно не согласен;
2 — отчасти не согласен;
3 — не знаю ответа;
4 — согласен;
5 — полностью согласен.

**Table:** Стимульный материал

№ п/п	Утверждение	1	2	3	4	5
1	Я часто прибегаю к ретуши собственных фотографий, прежде чем выкладываю их в сеть Интернет.					
2	Я предпочитаю пребывание в сети интимному общению с партнером.					
3	Я считаю, что оскорбления и нецензурная брать в Интернете – это нормально.					
4	Мне приятнее смотреть изображения или видео в Интернете, чем читать текст.					
5	Я считаю, что в социальных сетях не стоит выкладывать фотографии, на которых заметны недостатки внешности.					
6	Интернет позволяет мне выразить себя.					
7	Я общаюсь в Интернете с людьми из других городов или стран.					
8	Бывает, что я захожу в социальные сети без намерения с кем-либо пообщаться.					
9	Я испытываю чувство вины, когда понимаю, что мой образ в реальном мире отличается от моего образа в социальных сетях.					
10	Я преувеличиваю значимость моих достижений в Интернете.					
11	Я терплю поражение в попытках сократить время, проводимое «онлайн».					
12	Мне нравится, что в Интернете можно полностью проявить себя.					
13	Иногда я указываю неверные данные о своей личности в Интернете.					
14	Я публикую фотографии важных событий своей жизни в социальных сетях.					
15	Я не всегда указываю истинные данные о своей личности, такие как имя, фотография, местоположение, при регистрации в социальных сетях.					
16	Я считаю, что посты в социальных сетях позволяют мне донести мои мысли до большого количества людей.					
17	Я состою в Интернете в группах, посвященных идеальной внешности.					
18	Я часто посещаю профили незнакомых людей в социальных сетях.					
19	Я считаю, что в Интернете необязательно соблюдать правила русского языка.					
20	К несчастью, достоинства человека в реальной среде часто остаются непризнанными, как бы он ни старался.					
21	Я произвожу впечатление успешного и привлекательного человека в социальных сетях.					
22	Игры в интернете или посещение социальных сетей помогает мне изменить настроение.					
23	Я чувствую пустоту, депрессию, раздражение, находясь не за компьютером.					
24	В Интернете гораздо удобнее совершать покупки.					
25	Я считаю, что в сети Интернет мой образ должен быть идеальным.					
26	Я проверяю электронную почту и открываю страницы в социальных сетях первым делом после пробуждения.					
27	Я считаю, что вполне допустимо выдавать себя за другого человека в Интернете.					
28	Интернет позволяет мне избавиться от скуки.					
29	Пользователи Интернета считают, что я более профессионален, чем есть на самом деле.					
30	Я считаю, что на фотографиях и видео в Интернете я выгляжу моложе и привлекательнее.					
31	Случалось, что я вступал в конфликты в социальных сетях, не идентифицируя свою реальную личность.					
32	У меня есть альтернативные страницы в социальных сетях, где я выдаю себя за других людей.					
33	Я считаю, что выгляжу на фотографиях более эффектно в сравнении с реальностью.					
34	Если я плохого мнения о человеке и мне не нравится его поведение в Интернете, то почти не стараюсь скрыть это от него.					
35	Я замечаю, что время, проводимое мной в Интернете, увеличивается.					
36	Пользователи Интернета думают, что я умнее, чем есть на самом деле.					
37	Я не хожу в библиотеку, так как мне проще найти любую информацию в Интернете.					
38	Я часто листаю ленту новостей в социальной сети «просто так».					
39	Порой я чувствую непреодолимое желание обновить страницу в социальной сети.					
40	Бывает, что настаиваю на своем при обсуждении в Интернете, даже когда не уверен в своей правоте.					
41	При использовании Интернета мое настроение улучшается.					
42	Свои достижения и успехи я обязательно освещаю в социальных сетях.					
43	Бывает, я бесцельно просматриваю чужие страницы в социальных сетях.					

Спасибо за сотрудничество!

### Virtual Identity of Social Media Users (VISMU) Test

*Full name _________________________________________ Age ___________________________*

*Date _________________________________________ Gender _____________________________*

*E-mail ________________________________________________________________________________*

*Instructions:* Here you have statements concerning certain aspects of your behavior, communication, and image on social media. Using the answer scale from 1 to 5, express your degree of agreement with each of the statements below. The degree of your agreement corresponds to the following indicators:

1 — strongly disagree;
2 — disagree;
3 — not sure;
4 — agree;
5 — strongly agree.

**Table:** Stimulus material

# of item	Statement	1	2	3	4	5
1	I often photoshop my pictures before posting them on the Internet;					
2	I prefer being online to being intimate with a partner;					
3	I think insults and foul language on the Internet are a matter of course;					
4	I enjoy watching pictures or videos on the Internet rather than reading a text;					
5	I believe you should not post photos on social media showing flaws in appearance;					
6	The Internet allows me to express myself;					
7	I communicate on the Internet with people from other cities or countries;					
8	Sometimes I use social media without intending to communicate with anyone;					
9	I feel guilty when I realize that my image in the real world is different from my social media image;					
10	I exaggerate the significance of my achievements on the Internet;					
11	I fail to reduce my time online;					
12	I like that I can fully express myself on the Internet;					
13	Sometimes I give incorrect information about my identity on the Internet;					
14	I post pictures of important events in my life on social media;					
15	I do not always indicate my true identity, such as name, picture, location, when registering on social media;					
16	I believe social media posts allow me to convey my thoughts to many people;					
17	I am a member of Internet groups dedicated to an ideal appearance;					
18	I often visit profiles of strangers on social media;					
19	I believe it is not necessary to follow the rules of the Russian language on the Internet;					
20	Unfortunately, the merits of a person in the real world often remain unrecognized, no matter how hard they try;					
21	I make the impression of a successful and attractive person on social media;					
22	Playing online or visiting social media helps me to change my mood;					
23	I feel empty, depressed, and irritated when I’m not connected to the Internet;					
24	It is much more convenient to make purchases on the Internet;					
25	I believe my image should be perfect on the Internet;					
26	The first thing I do when I wake up is check my email and open social media pages;					
27	I believe it is quite conceivable to take on a different persona on the Internet;					
28	The Internet allows me to get rid of boredom;					
29	Internet users think I am more professional than I really am;					
30	I believe I look younger and more attractive in pictures and videos on the Internet;					
31	I have entered into conflicts on social media without identifying my real identity;					
32	I have alternative pages on social media where I take on a different persona;					
33	I think I look more impressive in pictures than in reality;					
34	If I have a bad opinion of a person and do not like their behavior on the Internet, I hardly try to hide it from them;					
35	I notice that the time I spend on the Internet is increasing;					
36	Internet users think I am smarter than I really am;					
37	I do not go to the library, as it is easier for me to find any information on the Internet;					
38	I often scroll through the news feed on the social media aimlessly;					
39	Sometimes I feel an overwhelming urge to refresh a page on social media;					
40	It happens that I insist on my own way when discussing on the Internet, even when I am not sure that I am right;					
41	My mood improves when I use the Internet;					
42	I am sure to highlight my achievements and success on social media;					
43	I aimlessly browse other people’s pages on social media.					

Thank you for your cooperation!

### The Key For Processing the Results of Virtual Identity of Social Media Users (VISMU) Test

*Full name __________________________________________ Age __________________________________________*

*Date ______________________________________________ Gender ________________________________________*

**Table:**

Cyberaddiction		Acceptance of Subculture		Virtual image	
Statement #	points	Statement #	points	Statement #	points
2		3		1	
4		6		5	
8		7		9	
11		12		10	
14		13		17	
16		15		21	
20		18		25	
22		19		29	
23		24		30	
26		27		33	
28		31		36	
35		32			
38		34			
39		37			
41		40			
42					
43					
**Sum of points on the scale**		**Sum of points on the scale**		**Sum of points on the scale**	
**Total points**					

### Test specification

When developing the specifications for the VISMU test, we defined the levels of virtual identity and its components. Interpretation and detailed description of different levels of the scales were based on content analysis of profiles of social network users with different kinds of virtual identity. When conducting a content analysis, we relied on an assessment of the specific content of the profile, and the features of the topics of the submitted posts, as well as the content of the graphic (photos) and text (signatures, comments) information.

#### CA scale

A high level (8–10 points) indicated an uncontrollable desire to use social media daily, generally without pursuing specific goals. We also observed a loss of interest in the real world combined with the increased importance of the virtual space of social media. There was a loss of control over the amount of time spent on social media, and a constant desire to use gadgets (smartphones, tablets, or computers). There was a desire to cover a wide range of life events on social media in a wide and detailed manner. The nature of users’ time spent on the Internet was aimless, and they had difficulty interacting with the real world. They experienced a change in mood depending on the possibility of using social media, a lack of critical attitudes to materials posted on social media, and the negative consequences of excessive spending time online.

A medium level (4–7 points) indicated the daily use of social media for a wide range of uses from communication to information searches. We observed the active use of the possibilities of social media, and users emphasized their advantages. Despite a significant amount of time spent on social media, control and criticality were preserved.

A low level (1–3 points) reflected purposeful, controlled, and non-systematic use of social media for specific tasks: informal or business communication, viewing news, etc. We observed selectivity in posting materials (pictures, videos, etc.) on social media. The advantages of real communication were emphasized. A selective attitude towards information posted on social media was noted.

#### AS scale

A high level (8–10 points) was characterized by the user’s expressed approval of the norms of the subculture of social media users in a wide range of situations. A low level of criticality combined with a fear of possible virtual sanctions was typical. The nature of Internet activities, as well as the user’s opinions, judgments, and perceptions, changed under the influence of these norms. In particular, users approved of otherwise unacceptable communication styles (use of foul language, rude and cynical expressions, cyberbullying); anonymity was acceptable (concealment or indication of false personal information); the use of written colloquial speech (characterized by tolerance to grammatical and punctuation errors) was allowed; and the possibilities of remote communication (with representatives of other cities, regions, countries) were used.

A medium level (4–7 points) reflected a partial acceptance of the norms of the users’ subculture mediated by the tasks of social media, and the specifics of a particular situation. The attitude towards these norms could change depending on the virtual sanctions imposed by reference users. The approval of some norms of the subculture and the rejection of other norms could be combined.

A low level (1–3 points) was characterized by a critical assessment of the norms of the subculture of social media users based on compliance with the individual’s value attitudes. The absence of a fear of virtual sanctions and a low dependence on the opinions and judgments of other social media users were typical. We observed independence in decision-making and defending one’s own opinion in the virtual space of social media.

#### VI scale

A high level (8–10 points) reflected the creation of an idealized self-image in the virtual space of social media, reflecting a complex of ideas about the user’s desired physical appearance and preferred psychological properties, through which the user enters into virtual communication. We observed digital enhancement before publishing pictures on social media and a critical attitude towards the shortcomings of people’s appearances as presented in the virtual space. The exaggeration of professional achievements, exaggerated demonstration of success and status in social media, and the creation of a virtual reputation were evident. In the case of significant discrepancies between the real and the virtual image, feelings of guilt might arise.

A medium level (4–7 points) was characterized by the creation of a virtual self-image reflecting a complex of physical and psychological personal properties partially corresponding to the real self-image. We observed the embellishment or concealment of some components of the created image for a more flattering self-presentation and effective communication on social media. The user emphasized the advantages of an easy transformation of the virtual image depending on the goals of using social media and also noted some possibilities for its idealization.

A low level (1–3 points) indicated the creation of a self-image in the virtual space of social media reflecting a complex of physical and psychological properties highly correlated (at the level of the capabilities of the social media interface) with the properties of the real self-image. We observed authenticity and stability in the user’s virtual image reflecting the genuine, unique, factual aspects of their real image (including ascriptive characteristics). Internet activities were carried out in the first person.

#### Integrated index

A high level (8–10 points) indicated the importance of social media for the user. We observed an increase in the value of online friends and building of virtual communication with a simultaneous decrease in the value of real communication. There was a lack of purpose for using social media. Reference users’ assessments on the individual’s values were important. Significant changes in the behavior or opinion, depending on the norms adopted in virtual communities, were evident. Manifestations of aggressiveness, including cyberbullying, were possible due to distorting or concealing personal data. We found attitudes towards a more effective presentation of physical characteristics and personal properties in social media underlying the creation of a virtual image aimed at demonstrating success and achievements on social media and creating a special virtual reputation. Playing alternative roles in the virtual space was possible. We observed the aimless use of time on social media and idealization in the creation of a virtual image, which performed a compensatory function.

A medium level (4–7 points) was characterized by the systematic use of social media, not limited to virtual communication. Although the time spent online was significant, the user showed criticality toward the negative consequences of excessive immersion in virtual space. Depending on the goals of using social media, the attitude towards the specific norms of the subculture adopted by the virtual communities changed. When creating a virtual image, both properties corresponding to the real self-image and idealized properties aimed at a more flattering self-presentation were demonstrated, which was determined both by the goals of using social media and the specifics of a particular situation. Although there were online friends and virtual communication, real interactions were still valuable to the user.

A low level (1–3 points) reflected the purposeful and conscious use of social media: communication, information search, and self-presentation. We observed a combination of a critical attitude towards the information posted on social media and the opportunities provided by them. The technologically determined limitation of the virtual space of communication was realized, which indicates more significance for real communication. The attitude towards the norms of the subculture of social media users was based on compliance with the individual’s values. The created virtual image generally reflected the user’s real self and was stable. We observed the purposeful use of social media and the creation of an authentic image.
